# Recent Advances in Wound Dressing

**Published:** 1889-03-09

**Authors:** 


### Within the Wards.

### RECENT ADVANCES IN WOUND DRESSING.

Anybody may be called upon at any moment to act as an amateur surgeon. An intelligent understanding of a few leading general principles will often prevent much pain and sorrow : sometimes it may even save life. The antiseptic system of surgery, with which the honoured name of Sir Joseph Lister is associated, is pretty generally understood even by the non-professional public. It essentially consists in keeping the atmospheric air and all the possible germs of disease which may be floating in it outside wounds. The air itself, if perfectly pure, will do no harm; but inasmuch as it is found impossible in practice to find an atmosphere of perfect purity, air is shut out of wounds because of the bad company it keeps. The Listerian method of surgery, although based on the soundest of principles and highly successful in practice, is both costly and troublesome. If the germs of possible disease can be excluded from wounds by any method which is simpler, less expensive, and more economical of time and labour, it is clear that a practical advance will be made in the surgical art. Mr. Jordan Lloyd, of Birmingham, has recently expounded his principles and methods in a paper which is full of interest both from a professional and a humane point of view. Mr. Lloyd believes in keeping a wound dry. The faith of ordinary mortals, and of many doctors, in some form of moist application for wounds is unlimited. Poultices, for example, are believed to be always safe and always suitable for any diseased surface. It is true that poultices are seldom actually dangerous, but they are very often highly injurious, and quite sufficient to prevent wounds from healing. The effect of a poultice is to make the part to which it is applied soft and "soppy." This is exactly what is wanted sometimes. But if anyone will put his toes or fingers into hot water for a couple of hours he will find them at the end of that time so "soppy" and sodden that the healing of a cut, a tear, or an ulcerated surface would be seen to be impossible. The natural fluids of a newly-made wound in a healthy part have a powerfully healing efficacy ; the less they are mixed with outside material of any kind, either fluid or solid, the more rapidly will healing take place. Of course in certain cases these fluids are present in excess, and then it is necessary to use something which may check or absorb the excess. But in an ordinary "cut" or laceration, if the bleeding can be stopped, and the edges of the wound brought accurately together, and fixed in that position, and then protected by a dry dressing and left quite motionless, that wound has been treated by a method which Nature herself entirely approves, and of which she shows her approval by a prompt cure.

In simple every-day language Mr. Lloyd explains how he has reached his present surgical standing-place. "In the year 1878," he says, "it was my privilege to be a house-surgeon under my late colleague, Mr. Sampson Gamgee ; and I need scarcely add that the principles which animated him in this matter of wound-treatment became familiar to myself. At that time 'rest,' 'position,' and 'pressure,' were extolled by him as the trinity of 'the Surgical Graces.' 'Dryness,' and 'infrequency of dressing,' were his guiding rules in practice." Gamgee understood, as did Liston, Syme, Bell, Pott, and Hunter before him, those great primary and essential conditions under which injured tissues most rapidly heal, and his affection for old surgical authority would not allow him to be carried away, helter skelter, on the tide of Lister's carbolic sea. He appreciated what was sound in the Listerian method and accepted it at once. He smiled at the "theory" and jested at the "Spray," and how far he was right, the practice of many of our leading living surgeons bears ample evidence. Shortly before Gamgee's death, he included a fourth among his "graces"—that of "antisepsis," although he never attached the importance to it to which it is properly entitled. "Rest," "position," and "pressure," are potent factors for good in the treatment of all wounds, but greater than any of these is perfect "surgical cleanliness," another term for "perfect asepsis." The essentials of wound treatment, therefore, constitute a quartette, in order of importance, as follows : (1) surgical cleanliness, (2) practical immobilisation, (3) uniform pressure, (4) moderate elevation. "Dryness of dressings" is scarcely second in importance to any of these.

The cleansing of parts to be operated upon by washing, and the thorough washing of the hands of the operator and his assistants, with the strictest care in making and keeping clean all instruments, towels, lint, and other necessary appliances, are held by Mr. Lloyd to be of the very essence of good surgery. "Sponges," says he, "I never use." Why does he never use them? Because they so readily contract and retain both fluid and solid impurities. Many surgeons do use sponges, and in this they may properly enough be left to their own discretion. Some of them, however, always insist upon a set of entirely new sponges for each important operation. In the domestic circle it is a good rule never to use a sponge in dressing a wound. If this rule is departed from it is found in practice that the nursery or bed-room sponge is employed, and these are generally more or less charged with soap, dust, fluff and other impure matters gathered from the bed-room or nursery. At home the only safety lies in avoiding sponges altogether in wound dressing. A little perfectly clean lint, well washed in hot water before being used, and then after use thrown away or burnt, is always safest and always best. The essence of Lister's antiseptic surgery is cleanliness. Many a careful mother was an antiseptic wound dresser before Lister was born. She knew nothing of antiseptic theories, but she knew all about the practice of cleanliness, and so secured her successful results.

"The meddlesome inquisitiveness," continues Mr. Lloyd, "which directs us to expose all wounds within twenty-four or forty-eight hours, simply for the purpose of seeing how they are going on, is calculated to do a tremendous amount of harm. Not a few primary unions are spoilt by the practice. It has always appeared to me like to the child who sows seeds in her toy garden, and digs them up next day to see what progress they have made. Recent wounds should be dressed only in response to (one of) three demands—high temperature, quick pulse, or pain. If all are absent . . . satisfactory progress is being made, whatever theory would suggest to the contrary. In the absence of any of the above indications I leave wounds undisturbed from one to four weeks." The use of a paper of this kind is to show what provision Nature herself makes for the restoration of injured parts to normal health and function. The argument may be summed up in a few words. The best surgery is that which secures for Nature the fullest opportunities of exercising her own powers of healing.

---

Dr. Scoby, of Brussels, to whom had been awarded the prize for the best essay on the dietetic treatment of disease, offered by the Royal Academy of Medicine of Belgium, died a few days before the award was announced.

Of all domesticated animals, oxen are the most liable to tubercular disease. The human and the bovine races are pre-eminently tubercular ; but, it is said, we are not liable to infection from our cattle, because our natural temperature is lower, and the germ of bovine tuberculosis will not develop till the temperature is raised to the degree naturally existing in the animals, that is, 101 deg. to 103 deg. Fahrenheit.

